# Prebiotic RNA Synthesis by Montmorillonite Catalysis

**DOI:** 10.3390/life4030318

**Published:** 2014-08-05

**Authors:** Sohan Jheeta, Prakash C. Joshi

**Affiliations:** 1 NoR HGT&LUCA, 1 Scott Hall Crescent, Leeds LS7 3RB, UK; E-Mail: sohan7@ntlworld.com; 2 New York Center for Astrobiology and Department of Chemistry and Chemical Biology, Rensselaer Polytechnic Institute, Troy, NY 12180, USA

**Keywords:** catalysis, chirality, mineral salt effect, montmorillonite, prebiotic chemistry, RNA

## Abstract

This review summarizes our recent findings on the role of mineral salts in prebiotic RNA synthesis, which is catalyzed by montmorillonite clay minerals. The clay minerals not only catalyze the synthesis of RNA but also facilitate homochiral selection. Preliminary data of these findings have been presented at the “Horizontal Gene Transfer and the Last Universal Common Ancestor (LUCA)” conference at the Open University, Milton Keynes, UK, 5–6 September 2013. The objective of this meeting was to recognize the significance of RNA in LUCA. We believe that the prebiotic RNA synthesis from its monomers must have been a simple process. As a first step, it may have required activation of the 5'-end of the mononucleotide with a leaving group, e.g., imidazole in our model reaction (Figure 1). Wide ranges of activating groups are produced from HCN under plausible prebiotic Earth conditions. The final step is clay mineral catalysis in the presence of mineral salts to facilitate selective production of functional RNA. Both the clay minerals and mineral salts would have been abundant on early Earth. We have demonstrated that while montmorillonite (pH 7) produced only dimers from its monomers in water, addition of sodium chloride (1 M) enhanced the chain length multifold, as detected by HPLC. The effect of monovalent cations on RNA synthesis was of the following order: Li^+^ > Na^+^ > K^+^. A similar effect was observed with the anions, enhancing catalysis in the following order: Cl^−^ > Br^−^ > I^−^. The montmorillonite-catalyzed RNA synthesis was not affected by hydrophobic or hydrophilic interactions. We thus show that prebiotic synthesis of RNA from its monomers was a simple process requiring only clay minerals and a small amount of salt.

## 1. Introduction

RNA is proposed as an important biopolymer in early life on the Earth where it would have provided both catalysis and acted as a repository of genetic information [1,2,3,4]. The “RNA World” hypothesis for the origin of life proposes that RNA formed first and that the DNA-protein world evolved from it [4,5,6,7]. Most hypotheses of the origins of biological organization suggest that RNA with chain lengths in the range of 30–50 nucleotides is needed to initiate catalysis that makes a genetic system viable [8,9]. Recent studies by Kazakoy and Altman [10], and Turk *et al.* [11] have demonstrated that even small oligonucleotide chains can perform catalytic activity. Ferris and coworkers [12,13] have shown that the oligomerization of activated mononucleotides can be achieved by the use of a montmorillonite catalyst, which occurs naturally on Earth. The mononucleotides, formed by prebiotic processes on the primitive Earth, are likely to be present in racemic mixtures even though only d-ribose is present in naturally occurring RNA. The question is: how was chiral selection introduced into the prebiological system? We will present the results of the analysis of the products formed in the reactions of d,l-ImpA and d,l-ImpU on Na^+^-montmorillonite [14,15,16,17,18].

It is noted that a partial incorporation of sodium ions in the bilayer of montmorillonite is essential to the catalytic activity of clay minerals in our model system for RNA synthesis using phosphorimidazolides of the nucleosides [19]. The level of sodium chloride in the ancient surface ocean was 1.5–2.0 times higher than the present day level of 0.6 M, suggesting sufficient abundance of sodium ions [20,21,22]. Catalytic montmorillonites are also known to expand the layer spacing upon contact with water [23], indicating that the presence of hydrophilic and hydrophobic minerals may have an effect on the catalytic action of montmorillonite. Therefore, inorganic salts which tend to salt-out organic compounds from water (hydrophobic interactions) and salts, which show salt-in effects (hydrophilic interactions) were investigated to examine their role in the oligomerization process [24,25]. This review provides a comprehensive summary of one of the authors (PCJ) presentation on the significance of montmorillonite clay and mineral salts in prebiotic RNA synthesis at the “Horizontal Gene Transfer and the Last Universal Common Ancestor Conference” in the Open University, Milton Keynes, UK.

## 2. Experimental Section

### 2.1. General

Adenine, adenosine, d-5'-adenosine monophosphate (d-AMP), anhydrous sodium perchlorate (NaClO_4_), cesium chloride (CsCl), 2,2'-dithiodipyridine, guanidine, imidazole, lithium bromide (LiBr), lithium chloride (LiCl), lithium hydroxide (LiOH), lithium iodide (LiI), lithium perchlorate (LiClO_4_), potassium chloride (KCl), potassium iodide (KI), potassium perchlorate (KClO_4_), sodium bromide (NaBr), sodium iodide (NaI), sodium perchlorate (NaClO_4_), sodium sulfate (Na_2_SO_4_), triphenylphosphine, triethylamine (TEA), Trizma base (Tris) and d-5'-uridine monophosphate (d-UMP) were obtained from Sigma (St. Louis, MO, USA). l-adenosione-5'-monophosphate (l-AMP) and l-Uridine-5'-monophosphate (l-UMP) were obtained from ChemGenes Corp (Wilmington, MA, USA). Perchloric acid was purchased from Aldrich (St. Louis, MO, USA). Acetone, acetonitrile (CH_3_CN), N,N-Dimethyl-formamide (DMF), dimethyl sulfoxide (DMSO), ether, hydrochloric acid (HCl), potassium hydroxide (KOH) and sodium hydroxide (NaOH) were obtained from Mallinckrodt (Phillipsburg, NJ, USA). Ultrapure molecular biology grade water was obtained from USB Corp (Cleveland, OH, USA). Anion (NaCl) and magnesium chloride (MgCl_2_) were procured from J.T. Baker (Phillipsburg, NJ, USA), Montmorillonite (Volclay^®^ SPV-200) was a gift from the American Colloid Co. (Arlington Heights, IL, USA).

### 2.2. Analytical Methods

**Scheme 1:**
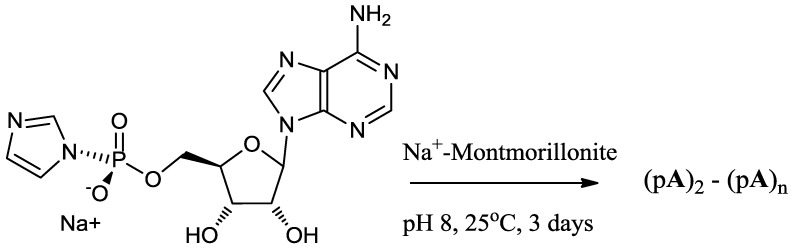
Model reaction of the phosphorimidazolide of adenosine on Na^+^-montmorillonite.

High-pressure liquid chromatography (HPLC) was performed on a Hitachi L-6200A intelligent pump system equipped with a Hitachi L-4000 UV detector operating at 260 nm. The negatively charged products were separated on a Dionex DNAPac^®^-100 (4.0 × 250 mm) analytical anion exchange column from Dionex Corporation (Sunnyvale, CA, USA) using a gradient of 0–0.4 M NaClO_4_ with 2 mM Tris at pH 8. The HPLC analysis of the combined extract of the reaction mixture to determine the length of oligomer was carried out on an ion exchange column using a gradient of 0–0.4 M NaClO_4_ with 2 mM Tris at pH 8 (Scheme 1). For the collection of a sufficient quantity of both dimer and oligomers, the separation of the oligomer mixture was carried out using a modified elution system consisting of a gradient of 2 mM–2 M ammonium acetate on the same ion exchange column. The separation profile of the oligomers with this reagent was not as good as observed with the NaClO_4_ and Tris reagent, but the presence of a huge amount of sodium perchlorate in the sample was avoided. The oligomers were collected by multiple injections. The sample was freeze dried, re-dissolved in 1 mL H_2_O and, if necessary, purified further by HPLC using same elution reagent. The re-purified and 5'-end dephosphorylated products were analyzed on a C-18 reverse phase column where they were resolved into multiple peaks.

### 2.3. Preparation of Catalytic Montmorillonite

Volclay^®^ (12 g) was treated with 0.5 M HCl (50 mL) by continuous stirring at 4 °C for 30 min. At the end of each treatment, excess acid was removed by centrifugation (3500 rpm) and decanting the supernatant. Fresh acid (50 mL) was added to the montmorillonite pellet and the treatment was repeated twice more. H^+^-montmorillonite was washed with 100 mL deionized water at 4 °C for 30 min with constant stirring. At the end of the washing, excess water was separated by centrifugation (3500 rpm) and decanting of the supernatant. Washing with water (100 mL) was repeated three times. The H^+^-montmorillonite slurry was added to water (1000 mL) and to this was added 45 mL of wet anion exchange resin to remove the residual HCl. The mixture was stirred for 30 min, pH was measured (3.00 ± 0.2), the anion exchange resin was removed by filtration using a stainless steel sieve (115 mesh) and the H^+^-montmorillonite slurry was removed and freeze dried. One gram of H^+^-montmorillonite was suspended in 100 mL of deionized water and was titrated with 0.02 M aqueous NaOH, LiOH or KOH to pH 7. The water was separated by centrifugation (3500 rpm) and the Li^+^, Na^+^ and K^+^-montmorillonite pellets were freeze dried.

### 2.4. Preparation of the Activated Nucleotide of AMP

**Figure 1:**
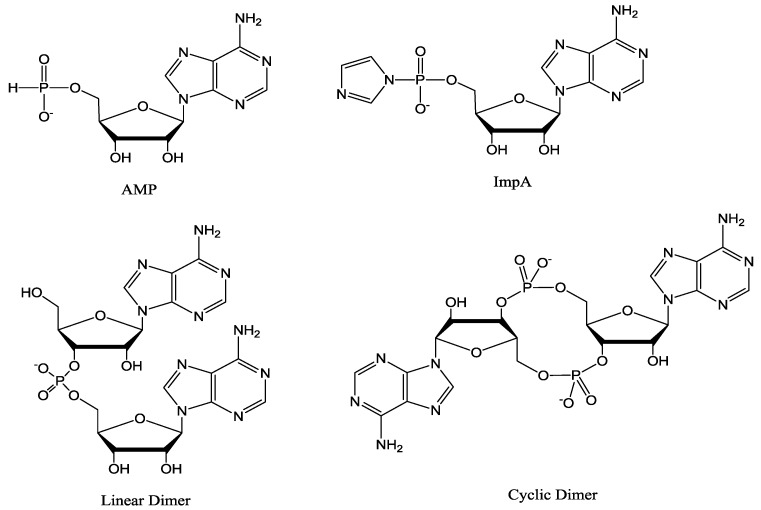
Structures of 5'-adenosine-monophosphate (AMP), 5'-phosphorimidazolide of adenosine (ImpA), linear and cyclic dimers.

A mixture of 5'-mononucleotide (AMP, free acid, 0.16 mmol, Figure 1) and imidazole (2.2 mmol) was dissolved in DMF (5 mL) in a 50 mL flask and the solvent was evaporated to near dryness at a reduced pressure. The evaporation was repeated twice with DMF (2 × 5 mL) to remove residual water. The residue was dissolved in DMF (2 mL) and DMSO (2 mL) and stirred with 2,2'-dithiodipyridine (0.64 mmol), triphenyl phosphine (0.46 mmol) and TEA (0.65 mmol) for 3 h. The resulting product was recovered from the clear yellow reaction mixture as a precipitate by adding the reaction mixture drop by drop to a solution of NaClO_4_ (0.35 g) in a mixture of ether (30 mL), acetone (20 mL) and TEA (2 mL), stirring at the same time. The stirring was continued for 2 h (to ensure complete formation of Na^+^ salt) and a colorless, flocculent solid was precipitated which was allowed to settle (15 min). The supernatant was decanted and the remaining reaction mixture was centrifuged. The resulting colorless pellet was washed twice with a mixture of ether (50 mL) and acetone (50 mL) and then with ether (2 × 50 mL) and dried overnight in a vacuum desiccator. Their purities were determined by reverse phase HPLC (purity > 99.9%).

### 2.5. Montmorillonite-Catalyzed Oligomerization of ImpA

Three sets of reactions were carried out:

***Reaction 1.*** d-ImpA (15 mM, Figure 1) on Na^+^-montmorillonite both with and without salts;
***Reaction 2.*** d-ImpA with d-ImpU (Total 15 mM) on Na^+^-montmorillonite; and
***Reaction 3.*** d,l-ImpA with d,l-ImpU (Total 15 mM) on Na^+^-montmorillonite.

The stock solution of activated nucleotide (15 mM) was prepared either in H_2_O, 1 M NaCl, a mixture of NaCl (0.2 M) and MgCl_2_ (0.075 M) or other inorganic salts, usually 1M unless stated. ***For Reaction 1***, 200-µL reaction mixture was added to 10 mg Na^+^-montmorillonite, the suspension was vortexed and allowed to stand at 24 °C for a period of 72 h (Scheme 1). For ***Reactions 2 and 3***, stock solutions of d-ImpA, l-ImpA, d-ImpU and l-ImpU (15 mM each) were prepared in a 0.075 M magnesium chloride and 0.2 M sodium chloride reagent. The ImpA and ImpU concentrations were determined by quantitative HPLC analysis of 10^−5^ M samples on a reverse phase column. The d- and l- mixtures of activated nucleotides were prepared at a total concentration of 15 mM in 0.075 M magnesium chloride and 0.2 M sodium chloride reagent by mixing the correct volume of each solution calculated on the basis of HPLC analyses. For ***Reaction 2***, 200 µL of d-ImpA with equimolar d-ImpU were reacted with 10 mg Na^+^-montmorillonite. The total concentration of activated mononucleotides was 15 mM. For ***Reaction 3***, 200 µL of a racemic mixture of d,l-ImpA with equimolar d,l-ImpU were reacted with 10 mg Na^+^-montmorillonite. The total concentration of activated mononucleotides was 15 mM. The reaction mixtures were vortexed and allowed to stand at 25 °C for 3 days (Scheme 1). The supernatant was collected from reaction mixtures containing Na^+^-montmorillonite by centrifugation at 14,000 rpm for 8 min. Further reaction products were desorbed from Na^+^-montmorillonite by extracting four times (2 × 1 h, overnight and 1 × 1 h) with 200 µL each of 30% CH_3_CN in 0.1 M NaCl elution reagent. The extracts were collected by centrifugation and combined with the supernatant to give 0.9 mL of combined extracts. The combined extracts were diluted to 1.0 mL and filtered (Alltima 0.45 µm nylon syringe filter), adjusted to pH 4 with 1 M perchloric acid and incubated at 37 °C for 4 h to cleave any unreacted imidazole groups from the activated nucleotides. The samples were analyzed by HPLC using an ion exchange column. The individual products (dimer, trimer, tetramer and pentamer fractions) were separated from the combined extracts on an ion exchange column and analyzed further on a reverse phase column.

### 2.6. Reaction and Analysis in the Absence of Minerals

Control solutions of ***Reaction 1*** were prepared in an appropriate salt (200 µL) without Na^+^-montmorillonite. For control ***Reactions 2 and 3***, samples were prepared in a 0.075 M magnesium chloride and 0.2 M sodium chloride reagent (200 µL) without Na^+^-montmorillonite. The total concentration of activated mononucleotides was 15 mM. The mixtures were allowed to stand at 25 °C for 3 days. At the end of the reaction time, the mixture was diluted to 1.0 mL with the 30% CH_3_CN in 0.1 M NaCl reagent, adjusted to pH 4 with 1 M HClO_4_ and incubated at 37 °C for 4 h to cleave any unreacted imidazole group from the activated nucleotides. The sample was analyzed on a Dionex ion exchange HPLC column.

### 2.7. Enzymatic Hydrolysis of Reaction Products

Dephosphorylation of the oligomer fractions to cleave the 5'-terminal phosphate group was carried out by treating the samples (500 μL) with APH (0.5 units) at pH 8 and incubation at 37 °C for 1 h, prior to their analysis on a reverse phase HPLC column.

## 3. Results and Discussion

### 3.1. Significance of the Investigation

This investigation was carried out to examine whether the Na^+^-montmorillonite-catalyzed reactions of activated mononucleotides are able to generate RNA oligomers and also if the same system may facilitate chiral selection in a racemic mixture. This is an important consideration because if activated mononucleotides were formed by prebiotic processes, it is likely that both enantiomers were present on the primitive Earth as a racemic mixture [26,27]. This study on the role of montmorillonite clay mineral catalysis in the formation of RNA oligomers on the primitive Earth provides insight into the selectivity of this potential prebiotic catalyst.

### 3.2. Oligomerization of Activated Mononucleotide on Na^+^-Montmorillonite

**Scheme 2:**
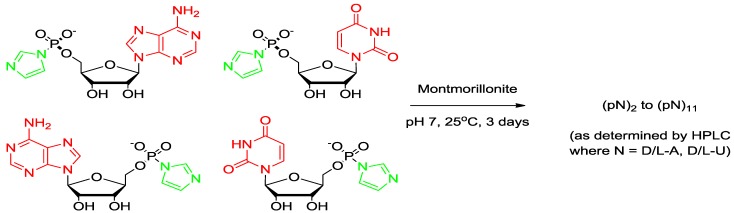
Reaction of the racemic phosphorimidazolides of adenosine and uridine on Na^+^-montmorillonite.

The oligomerization reaction of 15 mM activated d-mononucleotide on Na^+^-montmorillonite produced oligomers as long as 9-mers as determined by HPLC analysis on an ion exchange column (Scheme 1). The chain length of oligomers increased to 11-mer in racemic (d, l) reaction (Scheme 2), also see Figure 3. It has been established earlier that the increase in chain length of oligomers in a racemic reactions is largely due to the reduction in the yield of cyclic dimers [18]. Oligomerization reactions in the absence of Na^+^-montmorillonite resulted in the formation of no products beyond cyclic dimers and traces of linear dimers. The activated mononucleotides were hydrolyzed to their respective mononucleotides in >99% yield in the absence of Na^+^-montmorillonite. When the oligomerization of ImpA with Na^+^-montmorillonite was carried out in water, the cyclic dimers were the major reaction products (63.7%), with the linear dimers at 1.8% and traces of trimer were also detected (Table 1). The results showed that Na^+^-montmorillonite by itself is a poor catalyst. Earlier work has shown that highly acidic montmorillonite produced only pyrophosphates and AMP, whereas, alkaline montmorillonite produced mainly AMP. The longer oligomeric products were formed within a narrow window between pH 6–8 [28].

**Table 1:** A comparison of the homochirality between observed *versus* calculated values in a reaction mixture of d,l-ImpA with d,l-ImpU on Na^+^-montmorillonite.

Homochirality	Monomer	Dimer	Trimer	Tetramer	Pentamer
Observed	50%	63.5%	74.3%	92.7%	97.2%
Calculated	50%	50%	25%	12.5%	6.25%

### 3.3. Analysis of the Products of Reaction

**Figure 2:**
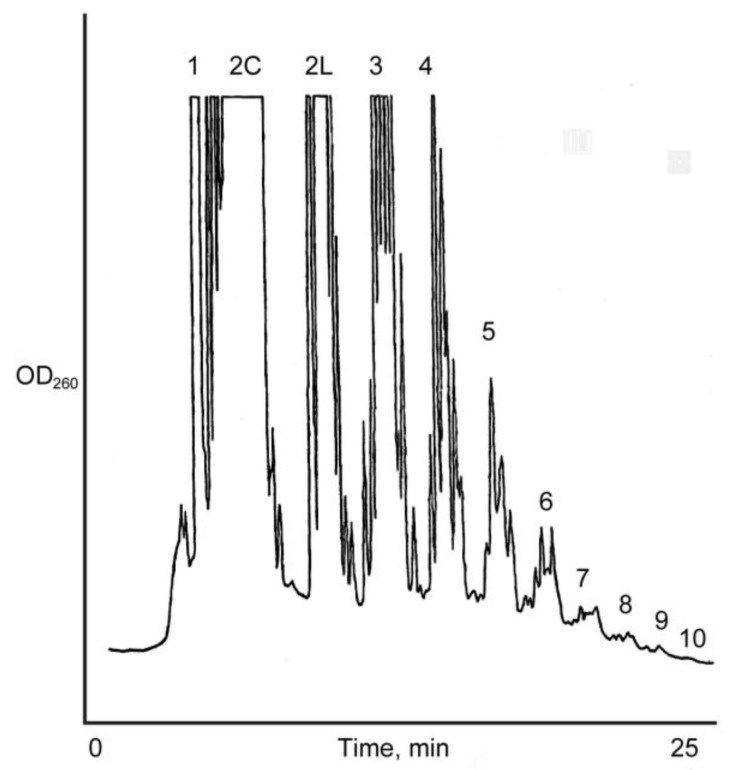
Anion exchange HPLC analysis of the Na^+^-montmorillonite catalyzed reaction of 15 mM D, l-ImpA with D, l-ImpU. Oligomer length up to 11-mer was detected. Cyclic (2C) and linear (2 L) dimers are eluted separately.

The identification and characterization of dimers and higher oligomers was based on the analysis of oligomers isolated from the reaction mixture of 15 mM activated mononucleotides on Na^+^-montmorillonite. This reaction produced oligomers up to 11-mers as detected by HPLC. However, for an accurate assessment of homochirality of dimers, an ideal condition was considered to be where no trimers are detected. Likewise, for the determination of the homochirality of trimers, an ideal condition will be where no tetramers are detected. Therefore, the Na^+^-montmorillonite catalyzed reactions of d,l-ImpA with d,l-ImpU were also carried out at a very low (0.15–4.8 mM) total concentration of the activated mononucleotides. All the dimers that were identified from a 15 mM reaction of activated mononucleotides were also detected at 0.15 mM concentrations and they followed a similar HPLC elution profile, as was observed with the dimers isolated from 15 mM reactions. Similarly, the trimers that were identified from the 15 mM reaction mixture were also detected at 1.5 mM concentration and they also followed a similar HPLC elution profile on a C-18 column. This result suggested that the relative ratios of dimers and trimers are independent of the initial concentration of the reactants. ***Reaction 2*** produced oligomers as long as 9-mers as determined by HPLC analysis on an ion exchange column. The chain length of oligomers increased to 11-mers in ***Reaction 3*** (Scheme 2 and Figure 2). The dimer fraction of ***Reaction 3*** after APH hydrolysis resulted in the detection of 18 peaks on the Alltima C-18 column. The major peaks were identified as uridine and adenosine. Of the remaining 15 peaks, 12 linear and three cyclic dimers were isolated and characterized. The homochirality of dimers was 63.5%. Out of the 16 trimers isolated, 10 were homochiral with an overall homochirality of 74.2%. The tetramers and pentamers were separated into 24 and 20 isomers, respectively. Their co-elution with those formed in ***Reaction 2*** revealed 92.7% and 97.2% homochirality, respectively (Table 1).

### 3.4. Effect of Salts in the Oligomerization of ImpA with Na^+^-Montmorillonite

This study was designed to investigate the possible role of inorganic salts in montmorillonite-catalyzed RNA synthesis. Particular emphasis was given to sodium chloride as its level in the ancient surface ocean was about 0.9–1.2 M, which is 1.5–2.0 times the present level of 0.6 M [20,21,22]. In our model prebiotic RNA synthesis system, the oligomerization of activated mononucleotides takes place within the clay layers in the presence of sodium chloride without any involvement of protecting groups. According to our findings, monovalent cations are essential in the model reactions [29]. Na^+^-montmorillonite alone did not produce products beyond dimer. However, addition of sodium chloride (0.1–2.0 M) to the ImpA and Na^+^-montmorillonite mixture increased oligomer length by at least a factor of 5–10, as determined by HPLC. Interestingly, the optimum oligomer chain length was detected at 1 M sodium chloride, which coincided with its abundance in the ancient oceans (0.9–1.2 M). Higher salinity reduced the formation of cyclic dimers, which are known to be a contributing factor in limiting the formation of preferred linear RNA. Magnesium chloride (0.075 M) produced oligomers up to 10-mers compared with small amount of dimers and traces of trimer formed without it. Sodium chloride (0.075 M) alone produced oligomers up to 6-mer under similar conditions. Magnesium chloride (0.075 M) and sodium chloride (1 M) together produced nearly the same outcome as those from sodium chloride (1 M) alone. The concentration of Mg^2+^ as combined MgCl_2_ and MgSO_4_ in the ocean today is approximately 0.05 M, which is about 8.3% of the current level of sodium chloride [30]. A similar ratio of these salts is likely to have been maintained in the ancient ocean. In this study, we have shown that although magnesium chloride enhanced the catalysis by Na^+^-montmorillonite, there was a similar effect when Na^+^ replaced the Mg^2+^. Therefore, Mg^2+^ is unnecessary, in contrast to what was previously thought. Therefore our model reaction system is now even more attractive, since it is even simpler. We now propose that saline clay mineral alone was needed to catalyze prebiotic RNA synthesis. A 1 M sodium chloride provided optimum oligomer length and product yield. The results from varying concentrations of sodium chloride are shown in Table 2. The yields of cyclic dimers (Figure 1) decreased from 76.9%–33.7% upon increasing the concentration of sodium chloride from 0.1 M–2 M. However, the hydrolysis of ImpA to AMP also increased from 12.9%–51.6% under similar conditions.

**Table 2:** Reaction of ImpA (15 mM) with Na^+^-montmorillonite (10 mg/200 μL) in the presence or absence of sodium chloride and/or magnesium chloride (72 h).

Reagent	Percentage Yield of Oligomers												
	I	IIc *	IIL **	III	IV	V	VI	VII	VIII	IX	X	XI	XII
*Effect of sodium chloride*													
2.0 M	51.6	33.7	9.32	3.15	1.40	0.62	0.12	0.05	0.03	T			
1.0 M	24.9	52.2	13.6	4.84	2.29	1.12	0.58	0.27	0.14	0.05	0.01		
0.2 M	15.8	68.5	12.0	2.68	0.79	0.21	0.02	T					
0.1 M	12.9	76.9	8.55	1.38	0.29	0.08							
*Effect of sodium chloride (1 M) and/or magnesium chloride (0.075* *M)*													
NaCl	21.1	58.8	12.6	4.06	1.89	0.96	0.29	0.14	0.10	0.06	T		
MgCl_2_	22.0	57.4	12.7	4.70	1.83	0.80	0.35	0.13	0.06	0.03	T		
*Effect of salt-in reagents* *(1 M)*													
LiClO_4_	15.3	60.3	11.4	5.75	3.13	1.73	1.20	0.62	0.33	0.17	0.05	0.03	
Guanidine	53.2	26.1	10.5	4.58	2.39	1.41	0.81	0.47	0.26	0.17	0.05	0.04	0.02
*Effect of salt-out reagents(1 M)*													
Na_2_SO_4_	11.4	60.5	13.0	6.17	3.36	1.93	1.40	0.81	0.52	0.40	0.32	0.19	T
LiCl	12.3	62.1	12.5	5.71	2.79	1.66	1.41	0.68	0.37	0.27	0.15	0.07	T
*Effect of reagents with no salt-in or salt-out effect* *(1 M)*													
LiBr	12.5	60.3	11.8	6.15	3.44	1.97	1.55	0.92	0.54	0.39	0.23	0.13	0.08
*Effect of no salts*													
H_2_O	34.5	63.7	1.80	T									

* Cyclic dimer; ** Linear dimer; T = trace.

### 3.5. Effect of Hydrophilic and Hydrophobic Salts in the Oligomerization of ImpA Catalyzed by Na^+^-Montmorillonite

Our previous studies with clay minerals revealed that the catalytic clays always swell in water, whereas, non-catalytic clays by and large do not have this property [19,28]. It is known that in the reactions carried out in an aqueous medium, the swelling of clay minerals is caused by the hydration of the exchangeable cations of the dry clay [23]. Therefore, Na^+^-montmorillonite catalyzed oligomerization reactions were carried out with mineral salts that tend to salt out organic compounds from water. Inorganic salts like sodium sulfate and lithium chloride did not show any preference for hydrophobic interactions in the Na^+^-montmorillonite catalyzed RNA synthesis. Similarly, salts that show salt-in effect (e.g., lithium perchlorate and guanidine) also showed no preference for hydrophilic interactions. Additionally, salts with no reported salt-in or salt-out effect such as lithium bromide did not produce any unusual outcomes (Table 2). This study clearly showed that a wide range of mineral salts might have contributed to the prebiotic RNA synthesis with no apparent bias for hydrophilic or hydrophobic interactions, although specific cations and anions may have had more impact than others.

### 3.6. Effect of Monovalent Cations and Anions in the Oligomerization of ImpA Catalyzed by Na^+^-Montmorillonite

The effect of cations upon the length of the oligomers was: Li^+^ > Na^+^ > K^+^. The yields of dimers (both linear and cyclic) as well as oligomers (III-XII, Table 3) were marginally higher with lithium chloride than with sodium chloride. Similar results were obtained with the perchlorate and iodide salts of Li^+^, Na^+^ and K^+^ [29]. In the reactions of ImpA with Na^+^-montmorillonite carried out separately in 1 M sodium chloride, sodium bromide and sodium iodide (Table 3), oligomers up to 11-mers, 10-mers and 9-mers, respectively, were detected. Their yields were marginally higher in sodium chloride compared with sodium bromide. Sodium iodide caused 48.1% hydrolysis of ImpA to AMP as compared to sodium bromide (23.9%) and sodium chloride (24.9%).

**Table 3:** Effect of cations and anions on the oligomerization of ImpA catalyzed by Na^+^-montmorillonite.

Oligomer Length →	Percentage Yield of Oligomers												
	I	II_c_*	II_L_**	III	IV	V	VI	VII	VIII	IX	X	XI	XII
*Effect of cations (1 M) with Chloride as a common ion*													
LiCl	12.3	62.0	12.5	5.68	2.79	1.65	1.41	0.68	0.36	0.29	0.22	0.12	T
NaCl	24.9	52.2	13.6	4.84	2.29	1.12	0.58	0.27	0.14	0.05	0.01	T	
KCl	72.2	21.2	5.29	1.10	0.34	0.05	0.02	T					
*Effect of anions (1* *M) with sodium as a common ion*													
NaCl	24.9	52.2	13.6	4.84	2.29	1.12	0.58	0.27	0.14	0.05	0.01	T	
NaBr	23.9	55.2	11.9	4.48	2.16	1.15	0.65	0.35	0.14	0.08	T		
NaI, 1M	48.1	39.5	7.95	2.56	1.07	0.52	0.23	0.05	0.02	T			

* Cyclic dimer; ** Linear dimer; T = trace.

### 3.7. Effect of Homoionic Salts in the Oligomerization of ImpA Catalyzed by Na^+^-Montmorillonite

The catalytic action of Li^+^-montmorillonite, Na^+^-montmorillonite and K^+^-montmorillonite with ImpA was investigated in aqueous solutions of 1 M lithium chloride, sodium chloride or potassium chloride, respectively (Table 4). The longest oligomers (11-mers) were detected in the reaction of ImpA with Li^+^-montmorillonite in 1 M lithium chloride. By changing the reagents in the ImpA and Li^+^-montmorillonite reaction mixture with 1 M sodium chloride or 1 M potassium chloride we observed reductions in chain lengths of oligomers to 10-mers and 6-mers, respectively. Likewise, in the reactions of ImpA with Na^+^-montmorillonite or K^+^-montmorillonite in 1 M lithium chloride, oligomers up to 11-mers and 9-mers in length were detected. In general, the yield of oligomers formed in both lithium chloride and sodium chloride was comparable and the degree of hydrolysis of ImpA to AMP was in the lower range (12%–25%). However, reactions carried out in potassium chloride led to considerable hydrolysis of ImpA (72%–73%) and oligomers only up to 6–7-mers were detected in relatively low yields.

**Table 4:** A comparison of the influence of homoionic salts with homoionic montmorillonite on the oligomerization of ImpA.

Oligo Length→ Salt Concentration ↓	Percentage Yield of Oligomers											
	I	II_c_*	II_L_**	III	IV	V	VI	VII	VIII	IX	X	XI
*Li*^+^-*Montmorillonite*												
LiCl, 1 M	16.0	60.0	12.6	5.47	2.74	1.38	0.97	0.45	0.23	0.13	0.03	T
NaCl, 1 M	20.1	58.9	11.7	4.32	1.95	1.00	0.55	0.25	0.13	0.10	T	
KCl, 1 M	72.6	21.5	4.73	0.95	0.17	0.05	T					
*Na*^+^-*Montmorillonite*												
LiCl, 1 M	12.5	63.0	12.3	5.56	2.77	1.64	0.92	0.62	0.34	0.21	0.14	T
NaCl, 1 M	24.9	52.2	13.6	4.84	2.29	1.12	0.58	0.27	0.14	0.05	0.01	T
KCl, 1 M	72.0	21.2	5.29	1.10	0.35	0.04	0.02	T				
*K*^+^-*Montmorillonite*												
LiCl, 1 M	13.2	63.2	12.2	5.39	2.54	1.59	0.97	0.48	0.25	0.18		
NaCl, 1 M	21.9	57.9	11.9	4.36	2.00	1.06	0.59	0.29	T			
KCl, 1 M	73.4	21.0	4.54	0.92	0.12	0.02	T					

* Cyclic dimer; ** Linear dimer; T = trace.

## 4. Conclusions

Montmorillonite catalyzed RNA synthesis and facilitated homochiral selection. The reaction depended on the salinity of the reaction medium. Optimum catalytic activity of Na^+^-montmorillonite was observed between 0.8–1.2 M NaCl, which resembles its concentration in the ancient oceans. Smaller cations (Li^+^ > Na^+^ > K^+^) and anions (Cl^−^ > Br^−^ > I^−^) showed increasing activity in producing longer oligomers.
